# Maximization of the Supportable Number of Sensors in QoS-Aware Cluster-Based Underwater Acoustic Sensor Networks

**DOI:** 10.3390/s140304689

**Published:** 2014-03-07

**Authors:** Thi-Tham Nguyen, Duc Van Le, Seokhoon Yoon

**Affiliations:** Department of Electrical and Computer Engineering, University of Ulsan, Ulsan 680-749, South Korea; E-Mails: nttham0611@gmail.com (T.-T.N.); anhduc.mta@gmail.com (D.V.L.)

**Keywords:** supportable number of nodes, QoS, optimization, underwater acoustic sensor network

## Abstract

This paper proposes a practical low-complexity MAC (medium access control) scheme for quality of service (QoS)-aware and cluster-based underwater acoustic sensor networks (UASN), in which the provision of differentiated QoS is required. In such a network, underwater sensors (U-sensor) in a cluster are divided into several classes, each of which has a different QoS requirement. The major problem considered in this paper is the maximization of the number of nodes that a cluster can accommodate while still providing the required QoS for each class in terms of the PDR (packet delivery ratio). In order to address the problem, we first estimate the packet delivery probability (PDP) and use it to formulate an optimization problem to determine the optimal value of the maximum packet retransmissions for each QoS class. The custom greedy and interior-point algorithms are used to find the optimal solutions, which are verified by extensive simulations. The simulation results show that, by solving the proposed optimization problem, the supportable number of underwater sensor nodes can be maximized while satisfying the QoS requirements for each class.

## 1. Introduction

As an emerging technique, underwater acoustic sensor networks (UASN) have a wide range of applications, such as oceanographic data collection, environment monitoring, undersea exploration, disaster prevention, assisted navigation and tactical surveillance [1–5]. In order to implement these applications, underwater nodes communicate with each other via acoustic channels that have unique characteristics, including the limited available bandwidth and a high and variable propagation delay [6–9].

In this paper, we consider a UASN that has a cluster-based network topology, in which each cluster is governed by a clusterhead (or gateway node), since it makes the network scalable and can readily provide network connectivity in a harsh communication environment [5,10–13]. In addition, the considered UASN consists of different types of underwater sensor nodes, some of which generate more important data than others, *i.e.*, the sensing data from some sensors may need to be delivered to the clusterhead with a higher PDR (packet delivery ratio). Therefore, the network needs to provide the sensor nodes with differentiated QoS (quality of service) in terms of PDR based on the QoS class to which the sensor nodes belong.

In such a network, an important problem is to maximize the number of nodes that the network can accommodate while still providing the required QoS for each class. In addition, as a related problem, when the operators deploy a UASN, they would want to know the achievable PDR value given the number of sensor nodes in the network. Intuitively, if the number of nodes in a UASN increases beyond a specific amount, the network may not be able to provide the demanded QoS, due to a high level of network traffic.

In order to address the problem of maximizing the supportable number of nodes, we focus on the MAC (medium access control) layer, since it plays a key role for providing QoS and dominates the overall performance of the network [14]. In particular, contention-based MAC protocols have received a lot of attention, due to the simplicity and applicability in UASNs [15–29]. Among various contention-based MAC protocols, Aloha-CS (Aloha with carrier sensing) is a potential low-complexity protocol for UASNs, since it offers a high throughput and low latency in a low network load without requiring time synchronization or a handshaking mechanism [18–20].

In this paper, we design a practical low-complexity QoS-aware MAC scheme and an optimization formulation for maximizing the supportable number of sensors in UASNs. We first estimate the packet delivery probability (PDP) in the MAC layer. Then, based on the PDP estimation, an optimization problem is formulated for maximizing the supportable number of sensors in a specific QoS priority class. The main idea of the formulation is to find optimal values of the maximum packet retransmissions for each QoS class, such that the number of nodes in a specific QoS class is maximized and every node can achieve the required QoS.

The custom greedy and interior-point algorithms are used to find the solutions to the optimization problem. Furthermore, extensive simulations are performed to verify the solutions. The simulation results show that our optimization formulation can maximize the supportable number of underwater sensor nodes, while satisfying the QoS requirement for each class.

The rest of this paper is organized as follows. Section 2 presents the related studies and compares them with the proposed scheme. The system model and problem definition are described in Section 3. Section 4 first discusses the packet delivery probability approximation, then describes the optimization problem formulation. We also discuss the approximation of the background traffic. The performance analysis using various scenarios is presented in Section 5, in which we also discuss solutions and the simulation setup. Finally, Section 6 concludes the paper.

## 2. Related Work

MAC protocols for UASN can be categorized into contention-free and contention-based protocols. The contention-free protocols include time division multiple access (TDMA), frequency division multiple access (FDMA) and code division multiple access (CDMA), in which different time slots, frequency bands or codes are assigned to different users to avoid collisions among transmissions.

FDMA divides the available frequency band into several sub-bands and assigns each sub-band to a node. Due to the limited available bandwidth of underwater channels, FDMA is not suitable for UASNs that consist of a large number of underwater sensors.

In TDMA, in order to avoid the collision of packets from adjacent time slots, guard times are added to the time slot. The high propagation delay in underwater acoustic communication channels requires long guard times, which limit the efficiency of TDMA [30]. Moreover, TDMA systems require precise synchronization for proper utilization of the time slots.

It is also known that CDMA-based protocols require a high complexity design for UASN. In addition, it is a challenging problem to assign pseudo-random codes to a large number of sensor nodes [2].

On the other hand, contention-based protocols have received significant attention for UASN, due to their simplicity, acceptable throughput and energy efficiency [15–23]. For example, the authors of [15] studied the performance of Aloha-based protocols in underwater networks and proposed two enhanced schemes that take advantage of the long propagation delay in the underwater acoustic channel and do not require handshaking or time synchronization.

It was also shown that, under the high and varying propagation delay in underwater acoustic channels, the performance of slotted Aloha becomes similar to that of pure Aloha [23]. The study in [16] proposed a propagation delay-tolerant Aloha protocol, where the authors address the space-time uncertainty by adding guard times to slotted Aloha.

Another simple yet practical Aloha-based protocol, Aloha-CS (Aloha with carrier sensing), was also studied and evaluated in [15,18–20]. In Aloha-CS, a node senses the carrier on the channel before it transmits data. The intended receiver sends an acknowledgment (ACK) packet to the source node to announce the successful reception. For unsuccessful transmissions, the retransmission mechanism with an exponential backoff can be also applied, *i.e.*, the data packet can be retransmitted up to a maximum limit of retries unless an ACK packet is received at the source node. According to the results presented in these studies, Aloha-CS (Aloha with carrier sensing) [18–20] can achieve high throughput and low latency without requiring time synchronization or handshaking.

The authors of [21] proposed an extension of the FAMA protocol [31] for UASN, namely slotted FAMA. Slotted FAMA is also based on carrier sensing and handshaking prior to data transmission. The new idea of slotted FAMA is that it uses time slotting to eliminate the requirement for excessively long control packets. The study in [22] proposed a reservation-based MAC protocol, T-Lohi, where a node sends a short tone to count the number of contenders. If it does not receive any other tones, it starts data transmission. Otherwise, it goes to the backoff mode.

Although our work is also based on channel contention, those studies differ from ours since they do not consider the provision of QoS or optimality.

There are few MAC protocols that address QoS provision in UASNs. However, there have been several MAC protocols that considered QoS provision for wireless sensor networks [32–39].

In particular, the authors of [37] proposed I-MAC, a hybrid TDMA/CSMA-based MAC protocol for wireless sensor networks. The I-MAC protocol is composed of two phases: the setup and transmission phases. During the setup phase, neighbor node discovery, slot assignment, local framing and global synchronization operations are successively performed. If a node owns assigned slots, it transmits data using those slots. If a node does not own any slot, it uses CSMA to access the channel. By using a different value of the CW (contention window), some groups of nodes can have a higher priority for accessing the channel.

As another example, the study in [38] proposed a MAC protocol that supports QoS in wireless sensor networks. It also uses a hybrid scheduling technique where dedicated time slots are assigned for data packet transmissions, and CSMA/CA-based random access periods are used for control packet transmissions. The MAC protocol consists of four phases: time synchronization, request for time slots, reception of slot schedules and data transfer.

However, the studies in [37,38] do not consider satisfying a given QoS requirement. In addition, they require tight time synchronization and overheads for slot requests and assignments. In contrast, the objective of our work is to design a low-complexity MAC scheme that supports differentiated QoS without requiring time synchronization or scheduling overheads.

There also have been attempts to design a QoS-aware MAC protocol based on channel contention for a wireless sensor network. For example, the study in [39] considered a transmitter-only network and proposed a MAC protocol to provide QoS using an optimal number of transmissions. That work also differs from ours, since it considered a network of nodes without an RFreceiver or packet queuing and a fixed number of transmissions of each packet in a given time interval. Moreover, the objective is different from that in our paper.

## 3. System Model and Problem Definition

**Figure 1.**
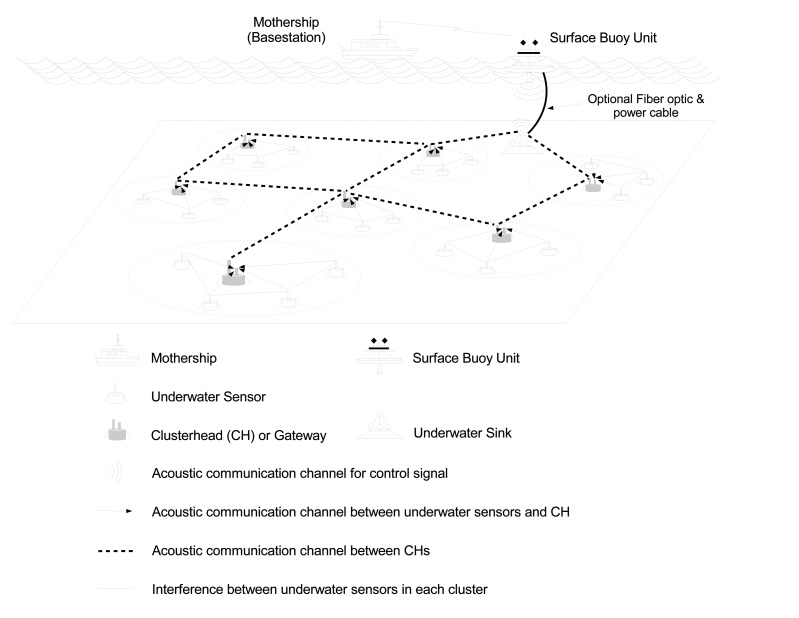
Cluster-based underwater acoustic sensor network.

In this paper, we consider a cluster-based UASN, where each cluster is governed by a clusterhead (or gateway node). As shown in Figure 1, each underwater sensor node (or, simply, U-sensor or node) belongs to one cluster. The clusterhead collects sensing data from U-sensors, performs data aggregation/fusion and then forwards the data to the underwater sink node. Clusterheads are equipped with two communication interfaces, so that they can use different channels for communicating with U-sensors and other clusterheads, respectively.

It is assumed that communications in a cluster do not interfere with communications in other clusters, due to the use of different carriers, and U-sensors transmit sensed data to the clusterhead using a direct acoustic channel [40]. Assigning channels to adjacent clusters or nodes has been considered in several studies [41–45].

U-sensors in a cluster are classified into several QoS classes, each of which has a required packet delivery ratio (PDR). In this paper, required PDR values are used to determine QoS classes. Every node generates a data packet at a predetermined rate and transmits them to the clusterhead. U-sensors in each QoS class are allowed to retransmit each data packet up to the maximum number of retransmissions, unless they receive the corresponding ACK packet from the clusterhead within the ACK timeout interval. Before a U-sensor transmits data, it first performs carrier sensing to assure that the channel is idle. It also performs exponential back-offs when collisions occur.

The considered optimization problem is the maximization of the number of nodes in a specific QoS class, which will be selected by the operators of the network, while providing the QoS for every node in each class.

In order to facilitate discussion, suppose that a set of *N* nodes in a cluster is divided into *m* QoS classes, (*Q*_1_,*Q*_2_,…,*Q_m_*), where class *Q_i_* contains *n_i_* nodes (1 ≤ *i* ≤ *m*). Nodes in each QoS class have a packet size, *s_i_*, and the corresponding packet transmission delay, $t_{d}^{i}$. Each node in class *Q_i_* is allowed to retransmit each data packet up to *x_i_* times and requires a minimum PDR of *p_i_*, where *x_i_* denotes the maximum number of retransmissions. Suppose also that class *Q_k_* is selected to maximize the number of nodes in the class, where 1 ≤ *k* ≤ *m*.

Therefore, in order to achieve the objective, while providing differentiated QoS to nodes, the core problem is to determine an optimal value of *x_i_* for each class, *Q_i_*, such that *n_k_* is maximized and every node in each class can achieve a PDR of at least *p_i_*.

## 4. Maximization of the Supportable Number of Sensors

In this section, we first describe the approximation of the packet delivery probability. Then, we present the formulation of the optimization problem. In addition, we discuss algorithms for finding solutions.

### 4.1. PDP Approximation

We first define the packet delivery probability (PDP) as the probability that a packet is successfully delivered at the clusterhead when it can be retransmitted up to *x* times.

In a UASN, the packet generation rate is usually low, due to the limited bandwidth. In such a network, very few packet losses result from the buffer overflow, since available space is likely when a new packet is generated. Consequently, PDP values can approximate PDR values in a UASN. Therefore, PDP is used in the optimization formulation for PDR.

Now, we discuss the approximation of the PDP of nodes in each class, *Q_i_*, where a node can retransmit a packet up to *x_i_* times. In order to approximate the PDP value, we first assume that the packet arrival in a UASN follows a Poisson process, which will also be verified in the following discussion. Then, the probability of *k* packet arrivals during an interval of time *t* is given by:

(1) $$P[n=k]=e^{-\lambda t}\frac{{\left(\lambda t\right)}^{k}}{k!}$$

where *λ* represents the arrival rate of background traffic in a time interval of *t* [46].

A U-sensor node in each class, *Q_i_*, transmits to the clusterhead a data packet in every interval, *T*. Suppose that a data packet arrives at the clusterhead at time *t*_0_ with the transmission delay of $t_{d}^{i}$. In order to avoid collisions for a packet that is transmitted from a node in class *Q_i_*, no packets from the other *N* – 1 nodes should arrive at the clusterhead during the interval $\left[t_{0}-t_{d}^{i},t_{0}+t_{d}^{i}\right]$, *i.e.*, there should be no packet arrival during the interval of $2t_{d}^{i}$.

Let $P_{s}^{i}$ and $P_{f}^{i}$ denote the probabilities of the successful and failed packet transmissions of a node in class *Q_i_* at the clusterhead, respectively, where $P_{f}^{i}=1-P_{s}^{i}$. Furthermore, let *λ_b_* denote the arrival rate of the background traffic for a node in an arbitrary class. Then, the probability that a data packet, which is transmitted from a node in class *Q_i_*, is successfully delivered at the clusterhead is given by:

(2) $$P_{s}^{i}=e^{-2\lambda_{b}t_{d}^{i}}$$

In order to verify the assumption of Poisson distribution of the packet arrival in a UASN, where a node performs carrier sensing and exponential back-offs, we conduct a simple simulation using Aloha and Aloha-CS protocols. The considered cluster in the network consists of 50 U-sensors and one clusterhead that are randomly deployed over an area of 1,555 m × 1,555 m. In this example, for simplicity, we assume that there is only one QoS class, *Q*_1_. Each U-sensor node is equipped with a half-duplex acoustic transceiver that has a data rate of 14 Kbps. Every U-sensor periodically generates a data packet of 160 bytes and sends it to the clusterhead. Each node is allowed to retransmit one data packet up to three times, unless it receives the corresponding ACK packet from the clusterhead. We calculate the probability of successful packet transmission in class *Q*_1_, $P_{s}^{1}$, according to Equation (2), and determine the actual successful individual packet transmission ratio from simulation. Then, we compare the value of $P_{s}^{1}$ from analysis and that from simulation.

**Figure 2.**
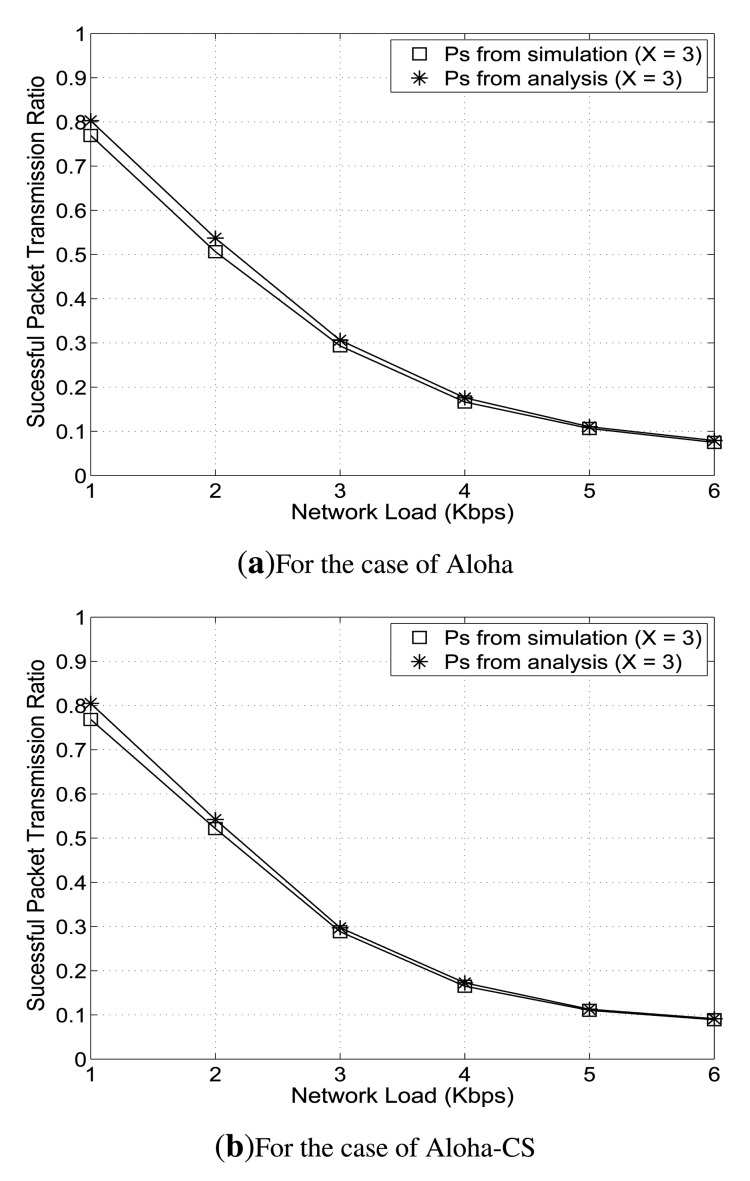
Approximation of the successful packet transmission ratio. Aloha-CS, Aloha with carrier sensing.

As shown in Figure 2, over different network loads from 1 Kbps to 6 Kbps, the approximation of $P_{s}^{i}$ is fairly similar to the actual successful individual packet transmission ratio. Therefore, in our work, we use the assumption that packet arrivals follow a Poisson process to design the optimization formulation. Now, we define $P_{s}^{i,j}$ and $P_{f}^{i,j}$ as the probabilities of the successful and failed delivery of the *j* -*th* transmission of a packet of nodes in class *Q_i_*, respectively. Furthermore, let *P*(*x_i_*) denote the PDP that the nodes in class *Q_i_* can achieve, and recall that one data packet can be retransmitted up to *x_i_* times. Then, *P*(*x_i_*) can be expressed as:

(3) $$P(x_{i})=P_{s}^{i,1}+P_{f}^{i,1}P_{s}^{i,2}+\ldots +P_{f}^{i,1}\ldots P_{f}^{i,x_{i}-1}P_{s}^{i,x_{i}}$$

Since each packet transmission can be regarded as an independent event based on the assumption of a Poisson process, $P_{s}^{i,j}=P_{s}^{i}$ and $P_{f}^{i,j}=P_{f}^{i}$ for all *j* (*j* = 1…*x_i_*). Therefore, *P*(*x_i_*) becomes:

(4) $$\begin{array}{ll} P(x_{i}) & =P_{s}^{i}\frac{1-{\left(P_{f}^{i}\right)}^{x_{i}}}{1-P_{f}^{i}}\\ & =1-{\left(P_{f}^{i}\right)}^{x_{i}}\\ & =1-{\left(1-e^{-2\lambda_{b}t_{d}^{i}}\right)}^{x_{i}} \end{array}$$

In the following section, we present an optimization formulation for maximizing the number of sensors in UASN, while satisfying the QoS requirement.

### 4.2. Optimization Problem Formulation

In this subsection, we describe the proposed optimization problem formulation that is a non-linear optimization problem.

Recall that the nodes in each class, *Q_i_*, need to guarantee their PDR requirement of at least *p_i_*. In other words, the approximated PDP of the nodes in each class needs to be at least *p_i_*. Specifically, the constraint function is expressed as:

(5) $$1-{\left(1-e^{-2\lambda_{b}t_{d}^{i}}\right)}^{x_{i}}\geq p_{i}$$

The actual arrival rate of background traffic for a node in an arbitrary class, *λ_b_*, is the total number of packet arrivals from the other *N* – 1 nodes in the time interval. It is a challenging problem to calculate the exact value of *λ_b_*, since the actual number of retransmissions for one data packet at a given time depends on the network traffic and status. Therefore, to simplify the problem, we use the maximum arrival rate of background traffic generated by all nodes in the network, *λ_max_*. In the following discussion, we prove that the required PDR can be satisfied by using *λ_max_*.

In order to calculate the value of *λ_max_*, we use the maximum number of retransmissions for each class, *Q_i_*, which is denoted by *x_i_*. Then, the maximum arrival rate of background traffic is given by:

(6) $$\lambda_{\text{max}}=\overset{m}{\underset{i=1}{\text{∑}}}\frac{n_{i}x_{i}}{T}$$

Then, the constraint function in which we use the maximum arrival rate, *λ_max_*, is given as:

(7) $$1-{\left(1-e^{-2\lambda_{\max}t_{d}^{i}}\right)}^{x_{i}}\geq p_{i}$$

#### Lemma 1

*Suppose that we use the maximum arrival rate of background traffic, λ_max_, to formulate the optimization problem. If we can determine an optimal value of x_i_ that satisfies the constraint function in Equation (7), then we can assure that x_i_ also satisfies the constraint function in Equation (5)*.

#### Proof

When we use the actual arrival rate of background traffic for calculating *P_s_*, then $P_{s}^{i}(\lambda_{b})=e^{-2\lambda_{b}t_{d}^{i}}$. Similarly, when we use the maximum arrival rate of background traffic to calculate $P_{s}^{i}$, then $P_{s}^{i}(\lambda_{\max})=e^{-2\lambda_{\max}t_{d}^{i}}$. From the fact that *λ_max_* ≥ *λ_b_*, we have $1-e^{-2\lambda_{\max}t_{d}^{i}}\geq 1-e^{-2\lambda_{b}t_{d}^{i}}$. Note that the value of *x_i_* is a positive integer. Therefore, we have the following relation:

(8) $$1-{\left(1-e^{-2\lambda_{\max}t_{d}^{i}}\right)}^{x_{i}}\leq 1-{\left(1-e^{-2\lambda_{b}t_{d}^{i}}\right)}^{x_{i}}$$

According to the constraint function in Equation (7), if we can find a value of *x_i_* that satisfies Equation (7), then the inequality $1-{\left(1-e^{-2\lambda_{\max}t_{d}^{i}}\right)}^{x_{i}}\geq p_{i}$ is always true. Combining the relation represented in Equation (8) and the constraint in Equation (7), we can achieve the following relation:

(9) $$p_{i}\leq 1-{\left(1-e^{-2\lambda_{\max}t_{d}^{i}}\right)}^{x_{i}}\leq 1-{\left(1-e^{-2\lambda_{b}t_{d}^{i}}\right)}^{x_{i}}$$

As a result, since *x_i_* satisfies the constraint function in Equation (7) in which the maximum arrival rate of background traffic, *λ_max_*, is used, then it also satisfies the constraint function in Equation (5) that uses the actual arrival rate of background traffic, *λ_b_*.

Therefore, we have the formulation of *P*(*x_i_*) for each class as follows:

(10) $$P(x_{i})=1-{\left(1-e^{-2\lambda_{\max}t_{d}^{i}}\right)}^{x_{i}}$$

Now, we describe our optimization problem formulation.

The objective of our optimization problem is to maximize the supportable number of nodes in a class, *Q_k_*, *n_k_*, where *k* is a given integer number from one to *m*. Note that *Q_k_* has the PDR requirement of *p_k_*. In order to maximize *n_k_*, we determine the relationships between *n_k_* and other variables. More specifically, from the fact that *P*(*x_k_*) ≥ *p_k_*, we have:

(11) $$1-{\left(1-e^{-2\lambda_{\max}t_{d}^{k}}\right)}^{x_{k}}\geq p_{k}$$

We replace *λ_max_* based on Equation (6) to show:

(12) $$1-{\left(1-e^{\frac{-2t_{d}^{k}}{T}\left(n_{1}x_{1}+\ldots +n_{k}x_{k}+\ldots +n_{m}x_{m}\right)}\right)}^{x_{k}}\geq p_{k}$$

From Equation (12), we obtain the following inequality:

(13) $$n_{k}\leq -\frac{1}{x_{k}}\left(\frac{T}{2t_{d}^{k}}\ln\left(1-{\left(1-p_{k}\right)}^{\frac{1}{x_{k}}}\right)+\overset{m}{\underset{\underset{i\neq k}{i=1}}{\text{∑}}}n_{i}x_{i}\right)$$

Then, the optimization formulation is that, given *n_i_* (*i* ≠ *k*) and *p*_1_, *p*_2_, …, *p_m_*, find *x*_1_,*x*_2_, …,*x_m_*, such that:

(14) $$\max\;\left[-\frac{1}{x_{k}}\left(\frac{T}{2t_{d}^{k}}\ln\left(1-{\left(1-p_{k}\right)}^{\frac{1}{x_{k}}}\right)+\overset{m}{\underset{\underset{i\neq k}{i=1}}{\text{∑}}}n_{i}x_{i}\right)\right]$$

subject to:

(15) $$\begin{array}{ll} {\left(1-e^{-2\lambda_{\max}t_{d}^{i}}\right)}^{x_{i}}-(1-p_{i})\leq 0, & i=1\ldots m \end{array}$$

(16) $$\begin{array}{ll} 1\leq x_{i}\leq l, & i=1\ldots m \end{array}$$

The constraint in Equation (15) is based on the requirement that the value of *x_i_* should guarantee *P*(*x_i_*) ≥ *p_i_*, where *P*(*x_i_*) is calculated according to the Equation (10). In addition, the value of *x_i_* is limited by an upper bound, *l*, as impressed in constraint Equation (16).

### 4.3. Finding Solutions

In order to find solutions to the proposed optimization formulation, we use a custom developed greedy algorithm and the interior-point method.

In the greedy algorithm, for each solution vector *x* = (*x*_1_,*x*_2_, …,*x_m_*), the maximum value of *n_k_* is first calculated by using Equation (13). If all PDR constraints are met using the vector and the value of *n_k_*, it stores those values and checks other vectors. Otherwise, *n_k_* is decremented until all constraint are satisfied. Among all possible *n_k_* values, the maximum is selected as $n_{k}^{\max}$, and the corresponding vector, *x*, is returned as a solution. The detailed algorithm is presented in Algorithm 1. Since there are *m* QoS classes, the vector of the optimum variable has *m* elements. Each *x_i_* can be one integer value from one to *l* (the upper bound of *x_i_*). Then, we have *l^m^* possible solutions. Furthermore, for each solution, up to *n_k_* times need to be evaluated. As a result, the worst-case computational complexity becomes *O*(*Ul^m^*), where *U* represents the upper bound of the node number in the system.

It is also worthwhile to note that even though the greedy algorithm seems to be expensive in terms of computational complexity, it may be affordable in a practical scenario. For example, when there are 3 QoS classes and *l* = 7, in most cases, less than 7,000 iterations are needed in our experiments, which is fairly acceptable, considering the computing power of modern computing systems.

In addition, the interior-point algorithm is used to find the solutions. The interior-point algorithm has been developed to solve linear or non-linear convex optimization problems with inequality constraints in a short amount time. The basic idea of this algorithm is to decompose the problem into a sequence of equality constrained problems and apply Newton's method to each problem [47]. There are a lot of variations of the interior-point method, and many of them have been shown to have a polynomial time complexity [48]. In this paper, we use the MATLAB optimization toolbox for the interior-point method with the assumption that each *x_i_* is a real number. Then, for simulation, we take the ceiling of *x_i_* after the solution is obtained, since the *x_i_* value should be an integer number in the real world. Note that, due to the real number relaxation and non-convexity of the objective function, it is possible that the solutions may not be the global optimal or may not even satisfy the required constrains. However, according to the simulation results, in most cases, the observed solutions are close to the global optimal values.



**Algorithm 1** The Custom Greedy Algorithm
**Inputs:***m*: number of QoS classes*T:* packet interval
$t_{d}^{i}(i=\{1\ldots m\})$: transmission delay in class *Q_i_**n_i_*(*i* ≠ *k*, *i* = {1…*m*}): number of nodes in each class except class *Q_k_**p_i_*(*i* = {1…*m*}): PDR requirement in class *Q_i_**l*: maximum number of retransmissions*U*: MAX_NODE (upper bound of the node number in the system)**Outputs:**The maximum supportable number of nodes 
$n_{k}^{\max}$ in class *Q_k_* and the corresponding optimal number of retransmission for each class 
$x_{i}^{\text{opt}}$1:*λ_max_* = 0;2:
$x_{i}^{\text{opt}}=0;$ ∀*i* = {1…m}3:*n_k_* = 0;4:
$n_{k}^{\max}=0;$5:**for** each (*x_i_*, …, *x_m_*) ∈ {1…*l*} **do**6: 
$n_{k}=\min\left(\left\lfloor -\frac{1}{x_{k}}\left(\frac{T}{2t_{d}^{k}}\ln\left(1-{\left(1-pk\right)}^{\frac{1}{x_{k}}}\right)+{\text{∑}}_{\underset{i\neq k}{i=1}}^{m}n_{i}x_{i}\right)\right\rfloor ,U-{\text{∑}}_{\underset{i\neq k}{i=1}}^{m}n_{i}\right);$7: **while**
$n_{k}>n_{k}^{\max}$
**do**8:   
$\lambda_{\max}={\text{∑}}_{i=1}^{m}\frac{n_{i}x_{i}}{T};$9:  
$P(x_{i})=1-{\left(1-e^{-2\lambda_{\max}t_{d}^{i}}\right)}^{x_{i}};$ ∀*i* = {1…m}10:  **if**
*P*(*x_i_*) ≥ *p_i_* ∀*i* = {1…*m*} **then**11:    
$n_{k}^{\max}=n_{k};$12:   
$x_{i}^{\text{opt}}=x_{i};$ ∀*i* = {1…*m*}13:   break;14:  **end if**15:  *n_k_* = *n_k_* − 1;16: **end while**17:**end for**18:**return** : 
$n_{k}^{\max}$, 
$x_{i}^{\text{opt}}$ ∀*i* = {1…m}


## 5. Performance Study

In this section, we first describe the simulation setup and then analyze the results of the simulations.

### 5.1. Simulation Setup

In order to evaluate the performance of the proposed protocol, we first consider a cluster with three QoS classes. Then, we extend our discussion to the case of four QoS classes. Finally, we consider a case where each QoS class has a different packet size.

When there are three QoS classes, the nodes in a cluster are partitioned into three QoS (in terms of PDR) classes (*Q*_1_,*Q*_2_,*Q*_3_), where *Q*_3_ is the selected QoS class in which we want to maximize the supportable number of sensors (*i.e.*, *k* = 3). It is assumed that the required PDR values for *Q*_1_ and *Q*_2_ are *p*_1_ = 0.95 and *p*_2_ = 0.8, while the PDR requirement for *Q*_3_ (*p*_3_) is a variable parameter. Furthermore, we suppose that the numbers of sensor nodes in classes *Q*_1_ and *Q*_2_ are five and 15, respectively (*i.e.*, *n*_1_ = 5,*n*_2_ = 15).

In case of four QoS classes, the nodes in the cluster are divided into four QoS classes, and *Q*_4_ is the selected QoS class (*i.e.*, *k* = 4). The required PDR values for *Q*_1_, *Q*_2_ and *Q*_3_ are *p*_1_ = 0.95, *p*_2_ = 0.9 and *p*_3_ = 0.8, respectively. The PDR requirement for *Q*_4_ (*p*_4_) is a variable parameter. The numbers of sensor nodes in classes *Q*_1_, *Q*_2_ and *Q*_3_ are five, 15 and 20, respectively.

In this paper, for practical simulation, we used the DESERTunderwater simulation framework [20], which incorporates spreading loss and various underwater noises, such as turbulence, shipping, wind and thermal noises. The observed solutions to the optimization formulation in Equations (14)–(16) are used as inputs for the simulations. The value of the maximum number of sensor nodes in the selected class is calculated using the solution. Then, this obtained value is also used for simulations as the number of nodes in the selected class.

Each node is equipped with a half-duplex acoustic transceiver that has a data rate of 14 Kbps and a transmission range of 1,100 m. The speed of the underwater acoustic signal is assumed to be 1,500 m/s. The data generation rate applies to every node in the network. The upper bound of the maximum number of retransmissions is set to seven.

### 5.2. Performance Analysis

In this subsection, we first present simulation results for a case with three QoS classes and discuss the results. Then, in order to show that our approach can support an arbitrary number of QoS classes, we extend our discussion to the case where a cluster has four QoS classes. Finally, we present the simulation results and analysis for a case where each of three QoS classes has a different packet size.

#### 5.2.1. Analysis of Results for Three QoS Classes

In this case, we consider a cluster that has three QoS classes. We first discuss the effects of the PDR requirement for a QoS class on PDR and on the maximum number of nodes in that QoS class. Then, we continue our discussion for the effects of the network load on the network performance. We assume that the PDR requirement of *Q*_3_ varies and the PDR requirements of *Q*_1_ and *Q*_2_ are given.

The effects of PDR requirement for class Q_3_:

In this case, every node transmits a data packet of 160 bytes to the clusterhead in every interval of *T* = 64 *s*, which leads to the transmission rate of 20 bps. The PDR requirement for class *Q*_3_ is varied from 0.7 to 0.86.

Tables 1 and 2 show the effects of the PDR requirement for class *Q*_3_ on the PDR and on the maximum supportable number of nodes in class *Q*_3_, when the greedy and interior-point algorithms are used, respectively. The tables show required PDR values (*P^req^*), a solution, *x*, calculated PDR values using optimal solutions (*P^anal^*), PDR values collected from simulations (*P^sim^*) and the maximum number of nodes in *Q*_3_ ( $n_{3}^{\max}$), calculated using the solution, which is also used for simulations.

As shown in Table 1, when the required PDR for class *Q*_3_ varies from 0.7 to 0.86, the maximum supportable number of sensor nodes in this class decreases from 84 to 50. The results indicate that, when the PDR requirement for the selected class decreases, the considered cluster in the network can accommodate a larger number of nodes, while satisfying the required PDR. For instance, if the PDR requirement for the nodes in class *Q*_3_ is 0.86, the considered cluster in the network can support 50 nodes in this class. However, if class *Q*_3_ is required to provide a PDR of 0.7, the considered cluster can support 84 nodes in class *Q*_3_. It is intuitive that the number of supportable nodes becomes greater as the required PDR decreases. However, one interesting point is that the supportable number of nodes is very sensitive to the PDR requirement. More specifically, when the PDR requirement of *Q*_3_ is lowered from 0.86 to 0.7 (e.g., 18.6% decrease), the supportable number of nodes in *Q*_3_ increases by approximately 68%.

Now, we discuss the selection of optimal *x* values to maximize the *n*_3_ and meet the requirements using examples in Table 1. As shown in Table 1, when $P_{3}^{\text{req}}$ is 0.76, the achievable $P_{3}^{\text{anal}}$ is only 0.761 with *x*_3_ = 2. This indicates that when $P_{3}^{\text{req}}$ becomes 0.78, $P_{3}^{\text{anal}}$ cannot meet the requirement any longer with *x*_3_ = 2, *i.e.*, 0.761 is less than 0.78. From Equation (11), there are two ways to increase $P_{3}^{\text{anal}}$ (suppose that *k* = 3). One way is to increase the value of *x_k_* in the left-hand side (lhs) of Equation (11). As *x_k_* increases, the lhs increases accordingly. Another way is to reduce *λ_max_*. It is clear that the lhs decreases as *λ_max_* decreases. It can be seen that, from Equation (6), the value of *λ_max_* depends on *x_i_*, where 1 ≤ *i* ≤ *m*.

Note that our greedy algorithm tests all possible cases. In this particular case, it appears that reducing *λ_max_* results in a larger *n*_3_, *i.e.*, *x*_1_ and *x*_2_ values are decreased to reduce *λ_max_*. This can be also regarded as follows. In order to increase $P_{3}^{\text{anal}}$, $P_{1}^{\text{anal}}$ and $P_{2}^{\text{anal}}$ are sacrificed by reducing *x*_1_ and *x*_2_. This also agrees with the results in Table 1 when $P_{3}^{\text{req}}$ is 0.78, $P_{1}^{\text{anal}}$ and $P_{2}^{\text{anal}}$ values have lower values with less *x*_1_ and *x*_2_ values than when $P_{3}^{\text{req}}$ is 0.76.

It is also possible in some cases that the greedy algorithm selects a higher *x*_3_ value with which a maximal *n*_3_ value can be obtained, while satisfying the requirements. For example, when $P_{3}^{\text{req}}$ values are varied from 0.82 to 0.84 in Table 1, the algorithm selects an increased value of *x*_3_ to maximize *n*_3_. In this case, *x*_1_ and *x*_2_ are also increased to meet $P_{1}^{\text{req}}$ and $P_{2}^{\text{req}}$, respectively. Note that when *x*_3_ increases, *λ_max_* also increases. However, in this case, the $P_{3}^{\text{req}}$ gain from raising *x*_3_ is higher than that lost from increasing *λ_max_*. Therefore, $P_{3}^{\text{req}}$ increases when a greater *x*_3_ value is used.

Another point to note is that when $P_{3}^{\text{req}}$ varies from 0.8 to 0.82, $n_{3}^{\max}$ also changes from 64 to 58, even with the same *x_i_* values. This is because $n_{3}^{\max}$ depends on $P_{3}^{\text{req}}$, as shown in Equation (13). Furthermore, note that $P_{1}^{\text{sim}}$, $P_{2}^{\text{sim}}$ and $P_{3}^{\text{sim}}$ values increase, since $n_{3}^{\max}$ has a lower value with the same *x_i_* values.

The results in Table 1 also show that, in all cases, both *P^anal^* and *P^sim^* are greater than *P^req^*, *i.e.*, the required PDR is always satisfied for all classes. This indicates that, by using the solution to the optimization formulation, the maximum number of nodes in a specific class can be obtained while satisfying the required PDR for all classes.

Table 2 shows the results based on the solution obtained using the interior-point algorithm. The results are close to those in Table 1, except that *x_i_* and $n_{3}^{\max}$ are real numbers. Recall that the ceilings of *x_i_* and the floor of $n_{3}^{\max}$ values are used for simulations. When the PDR requirement of class *Q*_3_ varies from 0.7 to 0.86, the maximum number of nodes in class *Q*_3_ decreases from 87 to 53.

Furthermore, note that, as shown in Tables 1 and 2, there are differences between *P^anal^* and *P^sim^* values, and in all cases, *P^sim^* values are greater than *P^anal^* values. In particular, we can observe these phenomena more clearly in Table 1, where there is no distortion, due to the ceiling effect. The reason for these phenomena is that the maximum arrival rate of background traffic, *λ_max_*, is used to calculate *P^anal^*, which results in a lower value of *P^anal^*. Therefore, this value can be considered as the lower bound of the PDR that can be achieved, and the results also agree with Lemma 1. In terms of $n_{3}^{\max}$, the greedy algorithm and interior-point algorithm show similar results, *i.e.*, interior-point algorithm outputs three more nodes on average. Note that the greedy algorithm shows a higher PDR for the highest priority group, *i.e.*, $P_{1}^{\text{sim}}$ with the greedy algorithm shows a 0.0091 higher value than with interior point algorithm. Since the greedy algorithm shows a comparable performance in terms of $n_{3}^{\max}$ and it shows a higher PDR, which is important for guaranteeing QoS, from now on, we focus on the results from the greedy algorithm.

##### Effects of Node Load

In this case, the PDR requirement for class *Q*_3_ is fixed to 0.7. Each underwater sensor periodically generates data packet of 160 bytes. Every node in the cluster transmits data at the rate from 20 bps to 50 bps to the clusterhead, *i.e.*, every node transmits data at every interval from *T* = 25.6 *s* to 64 *s*.

Table 3 shows the PDR requirements for each class ( $P_{1}^{\text{req}}$, $P_{2}^{\text{req}}$, $p_{3}^{\text{req}}$) and the various node loads in the network. It also shows the solution (*x*_1_, *x*_2_, *x*_3_) and the maximum supportable number of nodes in class *Q*_3_ ( $n_{3}^{\max}$) determined from the optimization formulation. The greedy algorithm is used to find the solutions in Table 3.

As shown in Table 3, all solutions have the same vector, *x*. Note that the node load is controlled by varying the packet transmission interval, *T*. Then, from Equation (13), it can be seen that *n_k_* is inversely proportional to the node load. In other words, *n_k_* and *T* are linearly dependent with given *p_i_* and *x_i_* values, which indicates that *n_k_* depends more on the change of the *T* value than on the change of *x_i_* values.

**Figure 3.**
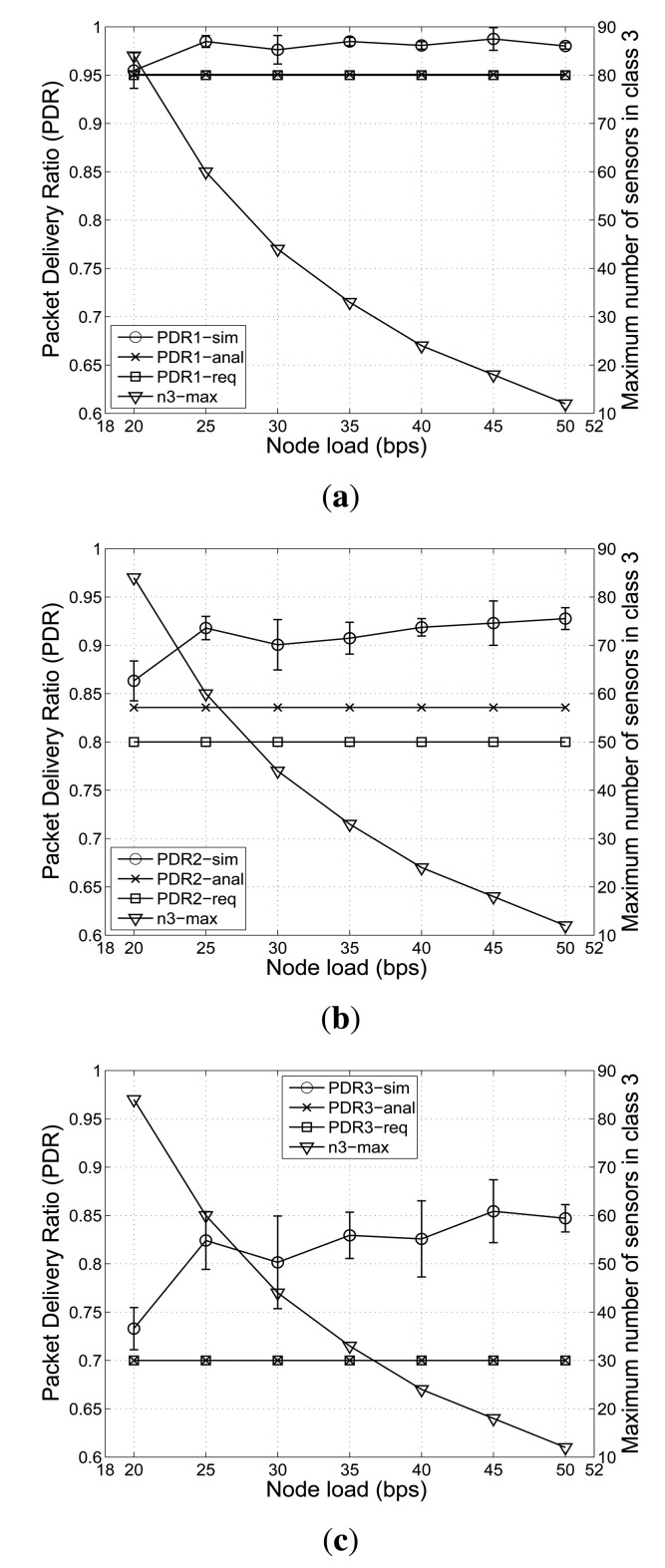
Effects of node load on the PDR (mean +/- standard deviation) achieved from the greedy algorithm and from simulations, and the maximum number of nodes in class *Q*_3_ with *n*_1_ = 5, *n*_2_ = 15. (**a**) For class *Q*_1_; (**b**) For class *Q*_2_; (**c**) For class *Q*_3_.

In order to facilitate understanding, we show and compare *P^req^*, *P^anal^* and *P^sim^* over different node loads in Figure 3. Furthermore, to show the confidence level of simulations, we present the standard deviation of *P^sim^* values along with mean values in Figure 3.

From Figure 3, we can see that, when the node load is high, a smaller number of nodes in class *Q*_3_ can be supported. On the contrary, when the node load becomes lower, the considered cluster in the network can accommodate a larger number of nodes in class *Q*_3_, while meeting the PDR requirements.

More specifically, Figure 3 shows that the nodes in all classes can satisfy their PDR requirements over various node loads. However, when the node load increases from 20 bps to 50 bps, the maximum supportable number of nodes in class *Q*_3_ shows a sharp decline from 84 to 12. In particular, up to the 30 bps node load, the supportable number of nodes in *Q*_3_ decreases sharply. Figure 3 also indicates that, by using the optimal value of the maximum number of retransmissions, the average PDR value of nodes in all classes are above their PDR requirements as the node load increases. For example, in Figure 3a, which shows obtained PDR values for class *Q*_1_, the average PDR values from both the analytical model and simulation are always equal to or greater than the required PDR value, 0.95. This also applies to class *Q*_2_ and class *Q*_3_ in Figure 3b,c, respectively.

#### 5.2.2. Analysis of Results for Four QoS Classes

In this section, we show that the proposed scheme can support four QoS classes. The greedy algorithm is used to find the solution in this experiment.

##### Effects of PDR Requirement for Class *Q*_4_

Similarly to the case of three QoS classes, every node transmits a data packet of 160 bytes to the clusterhead in every interval of *T* = 64 s, which leads to the transmission rate of 20 bps. The PDR requirement for class *Q*_4_ is varied from 0.7 to 0.86.

Table 4 shows the effects of the PDR requirement for class *Q*_4_ on the PDR values of the nodes in each QoS class, which are obtained from the greedy algorithm and simulations. It also shows the obtained maximum supportable number of nodes in class *Q*_4_ using the optimal *x* values.

As shown in Table 4, when the required PDR for class *Q*_4_ varies from 0.7 to 0.86, the maximum supportable number of sensor nodes, $n_{4}^{\text{max}}$, in this class decreases from 46 to 25 or, equivalently, the maximum supportable number of sensor nodes in the considered cluster decreases from 86 to 65. More specifically, if the required PDR for class *Q*_4_ is 0.7, the considered cluster can support 46 nodes in this class. On the other hand, if the PDR requirement for the nodes in class *Q*_4_ is 0.86, the cluster can support only 25 nodes in class *Q*_4_.

Note that, in some cases, $n_{4}^{\text{max}}$ remains the same even when $P_{4}^{\text{req}}$ increases. For example, when $P_{4}^{\text{req}}$ varies from 0.74 to 0.80, $n_{4}^{\max}$ keeps the value of 36. This is because the effect of the $P_{3}^{\text{req}}$ 's change is not enough for changing the *n_k_* value in Equation (13). Furthermore, $P_{3}^{\text{anal}}=0.83$ is sufficient for $P_{3}^{\text{req}}$ values from 0.74 to 0.80, which results in the same *x_i_* values and *n_k_*. The results also indicate that, among all feasible solutions, the solution *x* = {4,3,2,2} can achieve the maximum number of nodes in *Q*_4_ in the given $P_{4}^{\text{req}}$ range.

##### Effects of Node Load

**Figure 4.**
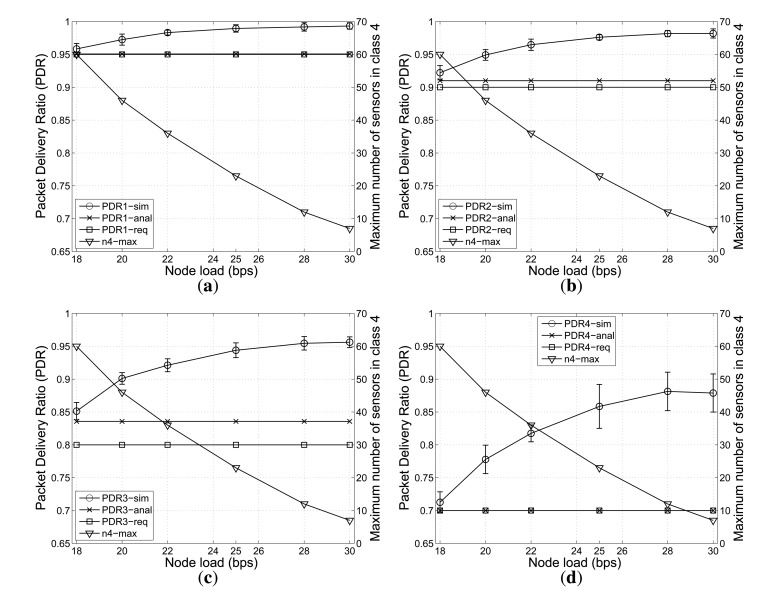
The effects of node load on the PDR (mean +/− standard deviation) achieved from the greedy algorithm and from simulations and the maximum number of nodes in class *Q*_4_ with *n*_1_ = 5, *n*_2_ = 15, *n*_3_ = 20. (**a**) For class *Q*_1_; (**b**) For class *Q*_2_; (**c**) For class *Q*_3_; (**d**) For class *Q*_4_.

In this case, the PDR requirement for class *Q*_4_ is fixed to 0.7. Every node in the cluster transmits data at the rate from 18 bps to 30 bps to the clusterhead with the data packet size of 160 bytes, *i.e.*, every node transmits data at every interval from *T* = 42.67 *s* to 71.11 *s*. Figure 4 compares *P^req^*, *P^anal^* and *P^sim^* over different node loads for all QoS classes.

As shown in Figure 4, the case of four classes shows a similar pattern to the three-class case. When the node load is small, a higher number of nodes in class *Q*_4_ can be supported. When the node load becomes higher, the considered cluster in the network can accommodate a smaller number of sensor nodes in class *Q*_4_, while satisfying the PDR requirements. Furthermore, the nodes in all classes can meet their PDR requirements over various node loads.

#### 5.2.3. Analysis of Results for Three QoS Classes with Different Packet Sizes

In this subsection, we consider a case where each QoS class has a different packet size. The sensor nodes in class *Q*_1_, *Q*_2_ and *Q*_3_ periodically generate data packets of 300 bytes, 200 bytes and 150 bytes, respectively. The PDR requirement for class *Q*_3_ is varied from 0.7 to 0.9. We discuss the effects of the PDR requirement for class *Q*_3_ on the maximum number of sensor nodes in class *Q*_3_.

From Table 5, we can see that the nodes in all classes satisfy their PDR requirements, *i.e.*, the *P^sim^* and the *P^anal^* values are greater than *P^req^* values. As the required PDR for class *Q*_3_ increases from 0.7 to 0.9, the maximum supportable number of sensor nodes in this class decreases from 51 to 27 nodes. The results also implicate that, in order to satisfy the required PDR for all classes, the maximum number of nodes in selected class decrease as the required PDR for this class increases.

Another point to note in Table 5 is that in many cases, $n_{3}^{\max}$ keeps the same value of 40. When $P_{3}^{\text{req}}$ is 0.74, the algorithm selects *x* = {6,2,2} with which the obtained values of $n_{3}^{\text{max}}=40$ and $P_{3}^{\text{anal}}=0.873$. When $P_{3}^{\text{req}}$ becomes 0.76, *n_k_* is calculated again using Equation (13). In this case, it appears that the effect of the $P_{3}^{\text{req}}$ 's change is not significant to change the new *n_k_* value. Moreover, $P_{3}^{\text{anal}}=0.873$ is sufficient for new $P_{3}^{\text{req}}=0.76$. Therefore, the *n_k_* remains at the same value. The phenomenon continues until $P_{3}^{\text{req}}$ becomes 0.86, which has a sufficient impact on changing the *n_k_* value in Equation (13). When $P_{3}^{\text{req}}$ varies from 0.88 to 0.9, $P_{3}^{\text{anal}}=0.895$ does not meet the new $P_{3}^{\text{req}}=0.9$. Therefore, as shown in Equation (11), *x*_3_ should be increased or *λ_max_* should be reduced. In this case, the algorithm chooses to reduce *λ_max_* by decreasing the *x*_1_ value from six to five, since it leads to a larger value of $n_{3}^{\max}$.

## 6. Concluding Remarks

In this paper, we have proposed a practical and low-complexity MAC scheme that does not require time synchronization or scheduling overhead, for QoS-aware and cluster-based underwater acoustic sensor networks (UASN). In particular, we have considered an optimization problem to maximize the supportable number of sensor nodes in UASNs that are required to provide differentiated QoS in terms of PDR. In order to address the problem, the packet delivery probability (PDP) has been estimated, and based on the estimation, an optimization formulation has been designed to determine optimal values of the maximum number of packet retransmissions for each QoS class. The greedy and interior-point algorithms are used to find the solutions, which are verified by simulations. The simulation results have shown that, by solving the proposed optimization formulation, the supportable number of underwater sensor nodes can be maximized, while satisfying the QoS requirements for each class.

## Figures and Tables

**Table 1.** The effects of the packet delivery ratio (PDR) requirement for class *Q*_3_ on the PDR achieved from the greedy algorithm and the maximum number of nodes in class *Q*_3_ (with *n*_1_ = 5, *n*_2_ = 15, *p*_1_ = 0.95, *p*_2_ = 0.80).

$P_{1}^{\text{req}}$	$P_{2}^{\text{req}}$	$P_{3}^{\text{req}}$	*Opt. Solut.*			$P_{1}^{\text{anal}}$	$P_{1}^{\text{sim}}$	$P_{2}^{\text{anal}}$	$P_{2}^{\text{sim}}$	$P_{3}^{\text{anal}}$	$P_{3}^{\text{sim}}$	$n_{3}^{\max}$

			*x*_1_	*x*_2_	*x*_3_							
0.95	0.80	0.70	5	3	2	0.951	0.965	0.836	0.875	0.701	0.729	84
0.95	0.80	0.72	5	3	2	0.959	0.970	0.852	0.896	0.721	0.750	78
0.95	0.80	0.74	5	3	2	0.965	0.977	0.868	0.908	0.741	0.774	72
0.95	0.80	0.76	5	3	2	0.972	0.982	0.883	0.919	0.761	0.803	66
0.95	0.80	0.78	4	2	2	0.960	0.972	0.800	0.850	0.800	0.819	64
0.95	0.80	0.80	4	2	2	0.960	0.972	0.800	0.850	0.800	0.819	64
0.95	0.80	0.82	4	2	2	0.967	0.977	0.820	0.866	0.820	0.847	58
0.95	0.80	0.84	5	3	3	0.953	0.981	0.840	0.913	0.840	0.904	55
0.95	0.80	0.86	5	3	3	0.962	0.986	0.860	0.927	0.860	0.919	50

**Table 2.** The effects of the PDR requirement for class *Q*_3_ on the PDR achieved from the interior-point algorithm, and the maximum number of nodes in class *Q*_3_ (with *n*_1_ = 5, *n*_2_ = 15, *p*_1_ = 0.95, *p*_2_ = 0.80).

$P_{1}^{\text{req}}$	$P_{2}^{\text{req}}$	$P_{3}^{\text{req}}$	*Opt. Solut.*			$P_{1}^{\text{anal}}$	$P_{1}^{\text{sim}}$	$P_{2}^{\text{anal}}$	$P_{2}^{\text{sim}}$	$P_{3}^{\text{anal}}$	$P_{3}^{\text{sim}}$	$n_{3}^{\max}$

			*x*_1_	*x*_2_	*x*_3_							
0.95	0.80	0.70	4.321	2.321	1.737	0.950	0.962	0.800	0.865	0.700	0.716	87.6
0.95	0.80	0.72	4.321	2.321	1.836	0.950	0.967	0.800	0.884	0.720	0.739	82.8
0.95	0.80	0.74	4.321	2.321	1.943	0.950	0.970	0.800	0.896	0.740	0.751	78.3
0.95	0.80	0.76	4.321	2.321	2.058	0.950	0.956	0.800	0.838	0.760	0.821	73.9
0.95	0.80	0.78	4.321	2.321	2.184	0.950	0.956	0.800	0.860	0.780	0.843	69.6
0.95	0.80	0.80	4.322	2.322	2.322	0.950	0.963	0.800	0.882	0.800	0.862	65.5
0.95	0.80	0.82	4.322	2.322	2.474	0.950	0.970	0.800	0.891	0.820	0.881	61.5
0.95	0.80	0.84	4.322	2.321	2.643	0.950	0.974	0.800	0.908	0.840	0.897	57.5
0.95	0.80	0.86	4.322	2.322	2.836	0.950	0.982	0.800	0.917	0.860	0.911	53.6

**Table 3.** The solutions from the greedy algorithm with various node loads (*n*_1_ = 5, *n*_2_ = 15).

*Node load* (*bps*)	$P_{1}^{\text{req}}$	$P_{2}^{\text{req}}$	$P_{3}^{\text{req}}$	*Opt. Solut*			$n_{3}^{\max}$

				*x*_1_	*x*_2_	*x*_3_	
20	0.95	0.80	0.70	5	3	2	84
25	0.95	0.80	0.70	5	3	2	60
30	0.95	0.80	0.70	5	3	2	44
35	0.95	0.80	0.70	5	3	2	33
40	0.95	0.80	0.70	5	3	2	24
45	0.95	0.80	0.70	5	3	2	18
50	0.95	0.80	0.70	5	3	2	12

**Table 4.** The effects of the PDR requirement for class *Q*_4_ on the PDR achieved from the greedy algorithm and the maximum number of nodes in class *Q*_4_ (with *n*_1_ = 5, *n*_2_ = 15, *n*_3_ = 20, $P_{1}^{\text{req}}=0.95$, $P_{2}^{\text{req}}=0.90$, $P_{3}^{\text{req}}=0.80$).

$P_{4}^{\text{req}}$	*Opt. Solut.*				$P_{1}^{\text{anal}}$	$P_{1}^{\text{sim}}$	$P_{2}^{\text{anal}}$	$P_{2}^{\text{sim}}$	$P_{3}^{\text{anal}}$	$P_{3}^{\text{sim}}$	$P_{4}^{\text{anal}}$	$P_{4}^{\text{sim}}$	$n_{4}^{\max}$

	*x*_1_	*x*_2_	*x*_3_	*x*_4_									
0.70	5	4	3	2	0.95	0.97	0.91	0.94	0.83	0.90	0.70	0.77	46
0.72	5	4	3	2	0.96	0.98	0.92	0.96	0.85	0.91	0.72	0.80	40
0.74	4	3	2	2	0.96	0.97	0.91	0.93	0.80	0.84	0.80	0.83	36
0.76	4	3	2	2	0.96	0.97	0.91	0.93	0.80	0.84	0.80	0.83	36
0.78	4	3	2	2	0.96	0.97	0.91	0.93	0.80	0.84	0.80	0.83	36
0.80	4	3	2	2	0.96	0.97	0.91	0.93	0.80	0.84	0.80	0.83	36
0.82	6	4	3	3	0.97	0.99	0.90	0.95	0.82	0.91	0.82	0.91	32
0.84	5	4	3	3	0.95	0.98	0.91	0.96	0.84	0.91	0.84	0.91	30
0.86	5	4	3	3	0.96	0.98	0.92	0.96	0.86	0.93	0.86	0.92	25

**Table 5.** The effects of the PDR requirement for class *Q*_3_ on the PDR achieved from the greedy algorithm and the maximum number of nodes in class *Q*_3_ (with *n*_1_ = 5, *n*_2_ = 15, *p*_1_ = 0.95, *p*_2_ = 0.80 and a different packet size for each QoS class).

$P_{1}^{\text{req}}$	$P_{2}^{\text{req}}$	$P_{3}^{\text{req}}$	*Opt. Solut.*			$P_{1}^{\text{anal}}$	$P_{1}^{\text{sim}}$	$P_{2}^{\text{anal}}$	$P_{2}^{\text{sim}}$	$P_{3}^{\text{anal}}$	$P_{3}^{\text{sim}}$	$n_{3}^{\max}$

			*x*_1_	*x*_2_	*x*_3_							
0.95	0.80	0.70	5	2	1	0.979	0.989	0.879	0.897	0.716	0.719	51
0.95	0.80	0.72	4	2	1	0.970	0.987	0.903	0.924	0.748	0.750	42
0.95	0.80	0.74	6	2	2	0.970	0.994	0.813	0.878	0.873	0.893	40
0.95	0.80	0.76	6	2	2	0.970	0.994	0.813	0.878	0.873	0.893	40
0.95	0.80	0.78	6	2	2	0.970	0.994	0.813	0.878	0.873	0.893	40
0.95	0.80	0.80	6	2	2	0.970	0.994	0.813	0.878	0.873	0.893	40
0.95	0.80	0.82	6	2	2	0.970	0.994	0.813	0.878	0.873	0.893	40
0.95	0.80	0.84	6	2	2	0.970	0.994	0.813	0.878	0.873	0.893	40
0.95	0.80	0.86	6	2	2	0.971	0.993	0.817	0.886	0.876	0.890	39
0.95	0.80	0.88	6	2	2	0.981	0.996	0.845	0.909	0.895	0.921	32
0.95	0.80	0.90	5	2	2	0.977	0.995	0.873	0.929	0.915	0.938	27
